# Translational insights into miR-126 and miR-423: biomarkers and therapeutic targets in cancer, cardiovascular, metabolic and kidney diseases

**DOI:** 10.3389/fmolb.2026.1813285

**Published:** 2026-04-22

**Authors:** Małgorzata Rodzoń-Norwicz

**Affiliations:** 1 Clinic of Internal Medicine, Nephrology and Endocrinology with Nuclear Medicine Laboratory and Dialysis Center, State Hospital 2 in Rzeszów, Rzeszów, Poland; 2 Department of Human Physiology, Faculty of Medicine, University of Rzeszów, Rzeszów, Poland

**Keywords:** biomarkers, cancer, cardiovascular disease, diabetic kidney disease, endothelial dysfunction, miR-126, miR-423, oxidative stress

## Abstract

MicroRNAs (miRNAs) are key post-transcriptional regulators that orchestrate complex gene regulatory networks controlling endothelial function, metabolic adaptation, inflammation, and tissue remodeling. Among them, miR-126-3p, miR-126-5p, and miR-423-5p have emerged as context-dependent modulators linking vascular biology with cardiometabolic and oncologic disorders. MiR-126, through its 3p and 5p strands, plays a central role in maintaining endothelial integrity and angiogenic homeostasis. By modulating phosphoinositide 3-kinase/protein kinase B (PI3K/AKT), mitogen-activated protein kinase (MAPK), and inflammatory signaling pathways, miR-126 regulates vascular repair, endothelial activation, and immune–vascular interactions. Reduced miR-126 expression is consistently associated with endothelial dysfunction, impaired angiogenic balance, and disease progression in diabetes, chronic kidney disease, and multiple cancers. In parallel, miR-423-5p regulates oxidative stress responses, transforming growth factor beta (TGF-β)–related pathways, and PI3K/AKT signaling in a context-dependent manner. Through modulation of redox balance, fibrotic remodeling, and cell survival pathways, miR-423-5p may exert either tumor-suppressive or pro-tumorigenic effects depending on cellular and microenvironmental conditions. In cardiometabolic and renal disorders, it contributes to microvascular dysfunction and inflammatory activation while also demonstrating translational potential as a circulating biomarker candidate. This review synthesizes shared and divergent signaling mechanisms governed by these miRNAs across disease states, emphasizing strand selection, target competition, and network-level cross-talk as determinants of context-specific outcomes. Understanding these multilayered regulatory interactions may support the development of network-oriented biomarker panels and precision RNA-based therapeutic strategies.

## 1 Introduction

The discovery of the first microRNA (miRNA), lin-4, in *Caenorhabditis elegans* in 1993, followed by the identification of let-7 in 2000, marked the beginning of a new era in molecular biology (Lee et al., 1993; Reinhart et al., 2000). These seminal findings demonstrated that small non-coding RNAs constitute integral components of gene regulatory networks and play a fundamental role in developmental timing and cellular differentiation.

Over the subsequent decades, miRNAs have been recognized as central regulatory elements governing gene expression across a wide range of physiological and pathological processes. Comprehensive mechanistic studies have revealed that miRNAs are deeply integrated into complex regulatory circuits controlling cellular homeostasis, stress responses, and disease progression (Shang et al., 2023). Their dysregulation has been associated with metabolic disorders, chronic inflammatory conditions, and systemic diseases, including diabetes and its complications (Al-Mahayni et al., 2023). The importance of microRNAs in gene regulation has been further highlighted by the 2024 Nobel Prize in Physiology or Medicine awarded for the discovery of microRNA-mediated regulatory mechanisms (Quévillon Huberdeau and Meister, 2025).

Beyond their involvement in disease pathogenesis, miRNAs are increasingly regarded as promising candidates for diagnostic biomarkers and therapeutic targets (Ho et al., 2022). In cancer, microRNAs regulate key hallmarks of tumor biology, including proliferation, apoptosis, angiogenesis, invasion, and metastasis (Budakoti et al., 2021). These observations have stimulated the development of miRNA-based therapeutic strategies and nucleic acid–based interventions aimed at restoring physiological gene expression networks (Liang and He, 2021).

Advances in high-throughput sequencing technologies and bioinformatic analyses have enabled the systematic identification and annotation of human miRNAs. According to miRBase release 22, more than 2,600 mature human miRNAs have been annotated, reflecting the remarkable complexity of miRNA-mediated regulatory landscapes (Kozomara et al., 2019).

High-throughput profiling approaches have further enabled global assessment of microRNA expression across tissues and disease states. Methods such as small RNA sequencing, microarray-based platforms, and RT-qPCR profiling panels allow large-scale identification and quantification of miRNAs in biological samples (Shang et al., 2023; Ho et al., 2022; Liang and He, 2021). More recently, emerging techniques including single-cell RNA sequencing and spatial transcriptomics have provided insights into cell-type–specific regulatory patterns (Shang et al., 2023). However, these approaches are associated with several limitations, including technical variability, differences in sample processing, challenges in data normalization, and difficulties in distinguishing functional causality from correlative expression changes. These methodological considerations should be taken into account when interpreting the role of miRNAs in human diseases.

It is estimated that microRNAs regulate more than 50% of human protein-coding genes, thereby influencing diverse signaling pathways and cellular processes (Shang et al., 2023). Their role in tumor biology and systemic disease mechanisms has been extensively documented (Smolarz et al., 2022).

At the molecular level, microRNAs are endogenous, single-stranded, non-coding RNA molecules approximately 19–23 nucleotides in length that regulate gene expression at the post-transcriptional level (Kim et al., 2025). They primarily exert their function by binding to complementary sequences within the 3′untranslated regions (3′UTRs) of target messenger RNAs, leading to mRNA degradation or translational repression (Kim et al., 2025). The tightly regulated process of miRNA biogenesis underlies their pleiotropic biological effects; however, as this topic has been extensively reviewed elsewhere, only key aspects relevant to their functional role are considered in this review.

Although thousands of microRNAs have been identified in the human genome, only a subset has demonstrated consistent translational relevance across multiple disease systems. Among these, miR-126 and miR-423-5p have attracted particular attention because of their involvement in endothelial homeostasis, cardiometabolic regulation, oxidative stress responses, and cancer-related signaling pathways. Notably, both microRNAs are detectable in circulating biological fluids and have been investigated as potential biomarkers in cardiovascular, metabolic, oncologic, and renal diseases.

This review does not aim to identify the most abundantly expressed or globally dominant microRNAs, but rather focuses on selected microRNAs with well-documented and complementary biological functions across multiple disease systems. In particular, miR-126 primarily reflects endothelial integrity and vascular homeostasis, whereas miR-423-5p represents a context-dependent regulator associated with cellular stress responses, fibrosis, and metabolic dysregulation. Their combined analysis enables a more integrative understanding of the interplay between vascular dysfunction and tissue remodeling.

The literature included in this review was identified through a structured search of PubMed, Scopus, and Web of Science databases. Articles published between 2020 and 2025 were considered. The following keywords were used: “miR-126”, “miR-423-5p”, “diabetic kidney disease”, “chronic kidney disease”, and “microRNA biomarkers”. Studies were selected based on their relevance to the biological functions and translational potential of miR-126 and miR-423-5p across major disease contexts. Priority was given to original experimental and clinical studies, while selected review articles were included to provide additional context and support interpretation of findings. Accordingly, this review focuses on miR-126 and miR-423-5p to provide an integrative overview of their biological roles and translational potential across major systemic diseases.

## 2 Biogenesis and mechanism of action of miRNAs

### 2.1 MiRNA biogenesis pathway

**FIGURE 1:**
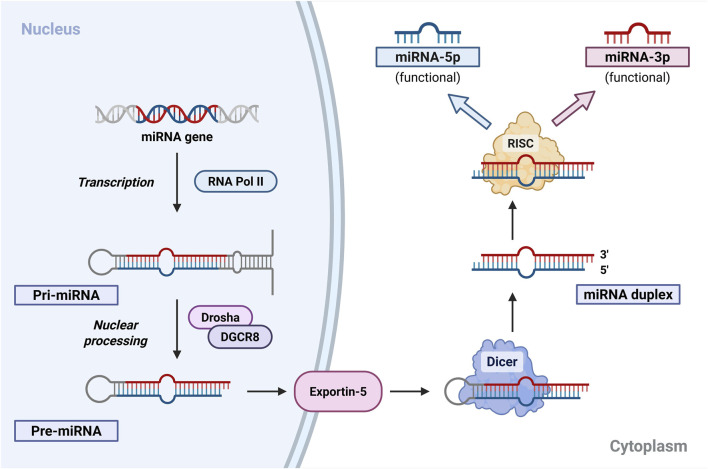
Canonical microRNA biogenesis and maturation pathway. miRNA genes are transcribed by RNA Pol II to generate primary transcripts (pri-miRNAs), which are processed by the Drosha–DGCR8 complex into precursor miRNAs (pre-miRNAs). Pre-miRNAs are exported to the cytoplasm via exportin-5, where Dicer cleaves them into ∼22-nucleotide miRNA duplexes. One strand is incorporated into RISC to form the mature functional miRNA, whereas the complementary strand may be degraded or remain active depending on cellular context. Mature miRNAs are designated as 5p or 3p according to their origin from the precursor hairpin. (Author’s own schematic illustration created with BioRender.com). Abbreviations: DGCR8 – DiGeorge syndrome critical region gene 8; Dicer – endoribonuclease Dicer; pri-miRNA – primary microRNA; pre-miRNA – precursor microRNA; RISC – RNA-induced silencing complex; RNA Pol II – RNA polymerase II.

miRNA biogenesis is a multistep process that generates mature regulatory RNA molecules from primary transcripts (Figure 1). MiRNA genes are transcribed by RNA polymerase II into primary miRNAs (pri-miRNAs), which are processed in the nucleus by the Drosha–DGCR8 complex into precursor miRNAs (pre-miRNAs) (Kim et al., 2025). These hairpin-structured molecules are exported to the cytoplasm, where Dicer cleaves them into ∼22-nucleotide double-stranded RNA duplexes (Shang et al., 2023; Kim et al., 2025).

One strand of the duplex is incorporated into the RNA-induced silencing complex (RISC), whereas the opposite strand is typically degraded (Kim et al., 2025). Importantly, both strands (miRNA-5p and miRNA-3p) may remain functionally active in a context-dependent manner (Shang et al., 2023; Kim et al., 2025). Strand selection depends on cellular context and contributes to the regulatory diversity of miRNA-mediated gene expression (Kim et al., 2025).

### 2.2 Mechanisms of gene silencing

**FIGURE 2:**
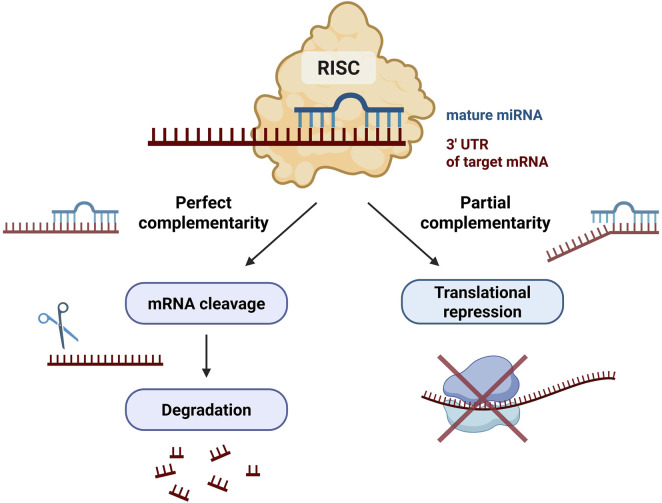
Mechanisms of microRNA-mediated gene silencing. Within the RISC, a mature miRNA binds to complementary sequences in the 3′UTR of target mRNAs. Near-perfect complementarity may induce endonucleolytic cleavage and mRNA degradation, whereas partial complementarity typically leads to translational repression and mRNA destabilization. These mechanisms enable post-transcriptional fine-tuning of gene expression. (Author’s own schematic illustration created with BioRender.com.) Abbreviations: miRNA – microRNA; mRNA – messenger RNA; RISC – RNA-induced silencing complex; 3′UTR – 3′untranslated region.

Within the RISC complex, mature miRNAs regulate gene expression by binding to complementary sequences in the 3′untranslated regions (3′UTRs) of target mRNAs (Figure 2) (Shang et al., 2023; Kim et al., 2025). Target recognition is primarily determined by the seed region of the miRNA (Kim et al., 2025).

In animal cells, near-perfect complementarity may result in mRNA cleavage, whereas partial complementarity typically leads to translational repression and mRNA destabilization (Shang et al., 2023; Kim et al., 2025). Through this mechanism, a single miRNA can regulate multiple target genes, and individual transcripts may be controlled by multiple miRNAs, forming complex regulatory networks (Shang et al., 2023). These mechanisms provide the basis for understanding the context-dependent roles of miR-126 and miR-423-5p in disease processes.

## 3 miR-126: biological role and clinical significance

### 3.1 Genomic context and biogenesis of miR-126-3p/5p

The MIR126 gene is located within an intron of the epidermal growth factor-like domain 7 (EGFL7) gene on chromosome 9q34.3 (Liu et al., 2022; Yang et al., 2022). Transcription of this locus gives rise to a precursor miRNA that is processed into two mature strands, miR-126-3p and miR-126-5p (Yang et al., 2022).

Rather than conforming to a strict guide–passenger paradigm, both strands may be functionally relevant depending on cellular context (Zeng et al., 2021; Yang et al., 2022). These isoforms differ in stability, target specificity, and biological activity. Unlike most miRNA duplexes in which one strand is preferentially degraded, both miR-126-3p and miR-126-5p have been shown to exert biological effects, particularly in endothelial regulation and in cardiometabolic and oncologic contexts (Zeng et al., 2021; Yang et al., 2022; Chen et al., 2021). Although genomic context, including intronic localization and co-expression with host genes, may influence microRNA expression patterns, target selection is primarily determined by sequence complementarity within the seed region and further modulated by cell-type–specific and disease-specific regulatory environments (Shang et al., 2023).

### 3.2 Molecular mechanisms, signaling pathways, and intercellular communication

MiR-126, particularly its isoform miR-126-3p, is abundantly expressed in endothelial cells, where it regulates angiogenesis, vascular integrity, and endothelial homeostasis (Zeng et al., 2021; Yang et al., 2022). Its activity involves key signaling pathways, including the phosphoinositide 3-kinase/protein kinase B (PI3K/AKT) pathway and the mitogen-activated protein kinase/extracellular signal-regulated kinase (MAPK/ERK) cascade, as well as vascular endothelial growth factor (VEGF)-dependent signaling, thereby supporting vascular repair and endothelial barrier function (Zeng et al., 2021; Yang et al., 2022; Di Paolo et al., 2021).

MiR-126-3p enhances pro-angiogenic signaling by directly targeting phosphoinositide-3-kinase regulatory subunit 2 (PIK3R2) and sprouty-related EVH1 domain-containing protein 1 (SPRED1), promoting endothelial proliferation and migration while limiting inflammatory activation (Yang et al., 2022; Di Paolo et al., 2021). Although miR-126-5p was initially considered a non-functional strand, accumulating evidence indicates that it contributes to oxidative stress regulation, apoptosis, and vascular remodeling in a context-dependent manner (Yang et al., 2022).

Importantly, miR-126 is not confined to endothelial cells but can also be detected in circulating extracellular vesicles and plasma, where it participates in intercellular communication and may serve as a circulating biomarker candidate in disease contexts (Yang et al., 2022; Abdipourbozorgbaghi et al., 2024). Altered circulating levels have been associated with disease progression in oncologic settings, supporting its potential role as a biomarker candidate (Sun et al., 2020; Abdipourbozorgbaghi et al., 2024; Grenda et al., 2024). Together, miR-126-3p and miR-126-5p orchestrate a complex molecular network linking metabolic, vascular, and oncogenic pathways. Their dysregulation disrupts angiogenic balance and contributes to the progression of chronic vascular and metabolic disorders (Yang et al., 2022).

### 3.3 miR-126 in cancer: tumor suppression and clinical implications

Aberrant miRNA expression contributes to the initiation and progression of human cancers (Budakoti et al., 2021; Smolarz et al., 2022). Among tumor-suppressive miRNAs, miR-126 has emerged as a frequently downregulated regulator in multiple malignancies (Yang et al., 2022; Chen et al., 2021). Both miR-126-3p and miR-126-5p have been reported to exhibit reduced expression in lung and other solid tumors, including breast and colorectal cancers (Yang et al., 2022; Chen et al., 2021).

Loss of miR-126 expression is associated with enhanced tumor proliferation, angiogenesis, metastatic potential, and resistance to therapy (Yang et al., 2022; Di Paolo et al., 2021; Chen et al., 2021). Mechanistically, miR-126 modulates oncogenic pathways such as PI3K/AKT and C-X-C chemokine receptor type 4 (CXCR4) by targeting PIK3R2 and other downstream effectors, thereby influencing tumor growth and vascular remodeling within the tumor microenvironment (Di Paolo et al., 2021; Chen et al., 2021).

The coordinated activity of both miR-126 strands (3p/5p) represents a dual regulatory mechanism contributing to endothelial stability and suppression of malignant transformation (Yang et al., 2022). The clinical relevance of miR-126 is further illustrated in organ-specific cancers discussed below.

#### 3.3.1 Lung cancer (LC)

Lung cancer is characterized by high malignancy and poor prognosis, with non-small-cell lung cancer (NSCLC) representing the predominant histological subtype. Due to nonspecific symptoms, lung cancer is often diagnosed at advanced stages, limiting surgical options.

Meta-analytical data indicate that reduced miR-126 expression is significantly associated with poorer overall survival and unfavorable clinicopathological features in NSCLC (Sun et al., 2020). Among tumor-suppressive miRNAs, miR-126 has therefore emerged as a clinically relevant regulator in lung cancer biology (Chen et al., 2021).

Several studies have demonstrated that serum miR-126-3p levels are significantly lower in patients with NSCLC compared with healthy controls, supporting its potential diagnostic value (Jiao et al., 2020). Reduced miR-126 expression correlates with higher Tumor–Node–Metastasis (TNM) stage and poorer prognosis (Jiao et al., 2020). In addition, circulating miRNA panels including miR-126 have shown promising diagnostic and prognostic performance across lung cancer cohorts (Abdipourbozorgbaghi et al., 2024).

Recent data indicate that miR-126-3p has been proposed as a biomarker and may also modulate therapeutic response. Circulating miR-126-3p has been associated with prediction of the effectiveness of immune-checkpoint inhibitor therapy in advanced NSCLC (Grenda et al., 2024), and it has been identified among key non-coding RNAs with potential biomarker relevance associated with NSCLC pathogenesis and progression (Kiełbowski et al., 2023). At the molecular level, downregulation of miR-126 (both 3p and 5p strands) in tumor tissue compared with adjacent non-tumorous tissue has been consistently observed (Liang et al., 2022). DNA methyltransferase 1 (DNMT1)-mediated hypermethylation of the epidermal growth factor-like domain-containing 7 (EGFL7) host gene contributes to silencing of miR-126 and promotes tumorigenesis (Liang et al., 2022). Aberrant methylation of the miR-126 locus further correlates with clinicopathological features of NSCLC (Mehrzad et al., 2024).

Constitutive activation of signal transducer and activator of transcription 3 (STAT3) is frequently detected in human cancers and correlates with adverse outcomes (Zou et al., 2020). MicroRNAs, including miR-126, have been shown to regulate components of the Janus kinase/signal transducer and activator of transcription (JAK/STAT) signaling axis, providing potential therapeutic opportunities (Sajjadi-Dokht et al., 2021). Circulating miR-126-3p has demonstrated potential independent diagnostic and prognostic value in NSCLC (Soliman et al., 2021). Functionally, miR-126-5p enhances radiosensitivity in lung adenocarcinoma cells by targeting enhancer of zeste homolog 2 (EZH2) via the Krüppel-like factor 2 (KLF2)/baculoviral inhibitor of apoptosis repeat-containing 5 (BIRC5) axis, promoting apoptosis and reducing clonogenic survival (Han et al., 2022). Moreover, miR-126 has been implicated in modulation of the PI3K/AKT signaling pathway in NSCLC (Wang Y. et al., 2023) and in the regulation of angiogenic cascades, including VEGF-dependent pathways (Lahooti et al., 2021).

Beyond intracellular signaling, miR-126 participates in broader tumor regulatory networks. Interactions between miRNAs and the lung and gut microbiota may influence tumor progression (Nucera et al., 2024). Furthermore, exosomal miR-126-3p has been identified as part of a circulating miRNA signature associated with NSCLC progression, supporting its potential role as a minimally invasive liquid-biopsy biomarker (Hassanin and Ramos, 2024).

Collectively, miR-126-3p and miR-126-5p cooperate in controlling proliferation, apoptosis, angiogenesis, radiosensitivity, and immunomodulation, underscoring their potential relevance as diagnostic, prognostic, and therapeutic biomarker candidates in NSCLC.

#### 3.3.2 Breast cancer (BC)

The important role of miR-126 in tumor biology has also been highlighted in studies on BC. In estrogen receptor–positive BC, miR-126 has been shown to decrease proliferation and mammosphere formation and to correlate with clinical outcome, supporting its tumor-suppressive relevance in this malignancy (Msheik et al., 2022). BC progression is characterized by aggressive invasion, early distant metastasis, and frequent multidrug resistance to standard chemotherapies, underscoring the need to better understand the molecular mechanisms underlying BC initiation and progression (Msheik et al., 2022).

One of the key mechanisms driving invasive BC is angiogenesis, primarily mediated by the VEGF family, particularly the VEGF-A isoform. VEGF-A is a secreted glycoprotein that binds to VEGF receptor tyrosine kinases and plays a pivotal role in endothelial proliferation and neovascularization (Wiszniak and Schwarz, 2021). Overexpression of VEGF-A correlates with tumor aggressiveness, poor prognosis, and enhanced metastatic potential, whereas suppression of VEGF-related angiogenic signaling by miR-126 contributes to reduced tumor invasiveness (Msheik et al., 2022; Wiszniak and Schwarz, 2021).

Another molecule strongly implicated in BC progression is A disintegrin and metalloprotease 9 (ADAM9), a membrane-anchored protease involved in cell adhesion, migration, extracellular matrix remodeling, and metastasis. Overexpression of ADAM9 promotes tumor invasion and is associated with worse clinical outcomes (Chou et al., 2020).

Jalil et al. comprehensively reviewed the emerging role of miR-126 as both a potential diagnostic and prognostic biomarker candidate and a therapeutic target in BC. Their analysis highlighted that miR-126 regulates angiogenesis, cell proliferation, and invasion, and interacts with targets such as ADAM9 and VEGF-A (Jalil et al., 2023).

Further mechanistic insight was provided by Sibilano et al., who investigated the intercellular crosstalk between platelets and BC cells. They demonstrated that platelets release microvesicles (MVs) containing miR-126-3p, which are internalized by BC cells and modulate oncogenic signaling. Among the key pathways influenced by platelet-derived miR-126-3p is PI3K/AKT pathway. AKT exists in three isoforms (AKT1, AKT2, AKT3); AKT1 mainly regulates proliferation and apoptosis, whereas AKT2 promotes cell migration, invasion, and metastatic potential. Elevated AKT2 activity is associated with poor prognosis in BC. MiR-126-3p directly targets AKT2, thereby suppressing the invasive potential of cancer cells and improving long-term survival (Sibilano et al., 2022).

Fu and Tong demonstrated that decreased miR-126-3p expression contributes to the development of trastuzumab resistance in human epidermal growth factor receptor 2 (HER2)-positive BC. Tumor cells resistant to trastuzumab exhibited reduced miR-126-3p levels and increased migratory capacity, whereas restoration of miR-126-3p resensitized cells to trastuzumab by inhibiting the phosphoinositide-3-kinase regulatory subunit 2 (PIK3R2)/AKT/mammalian target of rapamycin (mTOR) signaling pathway (Fu and Tong, 2020).

Although miR-126-3p is often considered the dominant functional strand, emerging evidence indicates that miR-126-5p may exert complementary tumor-suppressive effects in BC (Jalil et al., 2023). Preliminary findings suggest that miR-126-5p may influence epithelial–mesenchymal transition (EMT) and chemosensitivity through context-dependent regulation of oncogenic kinases within the PI3K/AKT and mitogen-activated protein kinase/extracellular signal-regulated kinase (MAPK/ERK) pathways (Jalil et al., 2023; Sibilano et al., 2022; Fu and Tong, 2020). This coordinated action of both miR-126 strands underscores the complexity of miR-126–mediated signaling and highlights their potential relevance for precision oncology (Jalil et al., 2023; Sibilano et al., 2022; Fu and Tong, 2020).

#### 3.3.3 Colorectal cancer (CRC)

CRC remains one of the most prevalent and deadly malignancies worldwide. Increasing evidence indicates that miR-126-3p expression is significantly altered in CRC and may have potential diagnostic relevance (Du et al., 2022). Most studies consistently report that miR-126-3p expression is significantly downregulated in CRC tissues compared with adjacent normal tissues or healthy controls (Du et al., 2022).

Reduced circulating levels of miR-126-3p have been associated with advanced disease stage and metastatic potential, particularly liver metastasis. Considering its remarkable stability in circulation, miR-126-3p represents a promising non-invasive biomarker candidate for CRC progression and prognosis (Ghorab et al., 2024). CRC develops through a multistep, multi-pathway process. Chronic inflammation is a critical mechanism promoting tumor initiation and progression, as it facilitates the emergence and clonal expansion of mutated epithelial cells. Inflammatory bowel diseases (IBD), such as Crohn’s disease and ulcerative colitis, are recognized risk factors for colitis-associated CRC (CAC). Various miRNAs, including miR-126, play essential roles in maintaining intestinal homeostasis and regulating inflammation-driven carcinogenesis (Wu et al., 2022).

Wu et al. demonstrated the protective role of miR-126-3p in CAC, showing that it directly targets the C-X-C motif chemokine ligand 12 (CXCL12) gene (Wu et al., 2022). CXCL12 interacts with its receptor CXCR4, forming a signaling axis that promotes macrophage recruitment, enhances interleukin-6 (IL-6) expression, and drives tumor progression. Elevated expression of the CXCL12/CXCR4 axis has been associated with poor prognosis in CRC patients (Ghorab et al., 2024; Wu et al., 2022). Importantly, upregulation of miR-126-3p suppresses CXCL12 expression, thereby reducing IL-6 production in macrophages and attenuating pro-tumor inflammatory signaling, collectively inhibiting tumorigenesis in CAC (Wu et al., 2022; Yang et al., 2023).

Yang et al. further emphasized the clinical significance of the CXCL12/CXCR4 axis and its central role in shaping the tumor microenvironment, highlighting its potential as a therapeutic target in cancer immunotherapy (Yang et al., 2023).

Together, these findings underscore that miR-126-3p exerts both anti-inflammatory and anti-metastatic effects in CRC, primarily by modulating the CXCL12/CXCR4/IL-6 signaling pathway.

#### 3.3.4 Hepatocellular carcinoma (HCC)

Among malignancies in which downregulation of miR-126-3p plays a crucial role, HCC stands out as one of the most aggressive and fatal cancers worldwide. Studies have demonstrated that miR-126-3p expression is significantly reduced in HCC tissues compared with adjacent non-tumorous liver tissue. Low miR-126-3p levels are associated with advanced tumor stage, increased invasiveness, and poor patient prognosis (Gondaliya et al., 2024).

In HCC, miR-126-3p has been shown to suppress tumor proliferation and invasion through regulation of oncogenic signaling pathways, underscoring its tumor-suppressive role in liver carcinogenesis (Gondaliya et al., 2024).

Further studies confirmed that miR-126-3p exerts antitumor effects in HCC by regulating the PI3K/AKT signaling cascade. Specifically, miR-126-3p inhibits PIK3R2, thereby reducing proliferation, invasion, and tumor growth in both *in vitro* and *in vivo* models (Du et al., 2014).

Therapeutically, miR-126-3p is also implicated in modulating resistance to sorafenib, a first-line systemic therapy for advanced HCC. Although sorafenib extends overall survival, its efficacy is often limited by acquired drug resistance. Tan et al. demonstrated that, in the context of sorafenib treatment, miR-126-3p downregulates SPRED1, resulting in activation of the extracellular signal-regulated kinase 1 (ERK1) signaling pathway and promoting sorafenib resistance. Conversely, overexpression of SPRED1 enhances the antitumor activity of sorafenib. Thus, targeting the miR-126-3p/SPRED1/ERK1 axis represents a promising strategy to overcome drug resistance and improve therapeutic outcomes in HCC (Tan et al., 2021).

### 3.4 miR-126 in diabetes mellitus (DM): metabolic and vascular implications

DM is currently one of the most prevalent metabolic diseases worldwide and represents a major global health burden. Despite advances in understanding its molecular mechanisms and improvements in treatment strategies, both incidence and DM-related mortality remain high. The mortality rate among patients with DM is substantially increased compared with non-diabetic individuals, largely due to the elevated risk of cardiovascular disease (CVD). Therefore, novel and reliable biomarker candidates for early diagnosis, disease monitoring, and therapeutic response are urgently needed. Circulating miR-126 has been proposed as a potential predictor of long-term mortality in patients with type 2 Diabetes Mellitus (T2DM), although current evidence is primarily based on meta-analytical data and remains subject to important limitations (Pordzik et al., 2021).

MiRNAs have emerged as critical regulators in the pathogenesis of DM and its complications, and they are increasingly explored as potential biomarkers and therapeutic targets. Benko et al. highlighted the role of miR-126-3p as a promising early diagnostic biomarker candidate for T2DM, demonstrating significantly lower serum levels in T2DM patients compared with healthy controls. Moreover, miR-126-3p exhibited high sensitivity and specificity, suggesting its potential application in future diagnostic tools (Benko et al., 2023).

Pramanik et al. reported a correlation between decreased serum and vitreous humor levels of miR-126-3p and the development of non-proliferative diabetic retinopathy (NPDR) in T2DM patients. Individuals with NPDR showed significantly reduced miR-126-3p levels compared to patients without diabetic retinopathy (NDR), supporting its potential diagnostic relevance in early microvascular complications (Pramanik et al., 2022).

A comprehensive meta-analysis, including 14 studies and 2,747 patients with diabetic nephropathy (DN), suggested that miR-126-3p is among the more consistently reported circulating biomarker candidates for this condition. Serum levels of miR-126-3p were consistently reduced, whereas urinary levels varied across studies, possibly reflecting differences in tubular reabsorption and urinary excretion at different disease stages (Jiang et al., 2025). These findings align with a literature review emphasizing the dual potential of miR-126-3p as both a biomarker candidate and therapeutic target in diabetic kidney disease (DKD) (Rodzoń-Norwicz et al., 2025).

Wang et al. further demonstrated that improved glycemic control in patients with diabetic ulcers (DU) increased serum miR-126-3p expression and enhanced wound healing, suggesting a link between miR-126-3p and tissue regeneration (Wang L. et al., 2023).

Physical exercise, a cornerstone of DM management, improves glycemic control and reduces cardiovascular mortality. Aerobic exercise has been shown to upregulate miR-126-3p expression and enhance angiogenic and endothelial signaling in diabetic myocardium (Dastah et al., 2020). Ma et al. revealed that chronic aerobic exercise elevates miR-126-3p expression in both serum and cardiac tissue, in proportion to exercise intensity, conferring cardioprotective effects through anti-inflammatory actions and improved endothelial function (Ma et al., 2022).

Finally, the importance of miR-126-3p in bridging metabolic and vascular pathology is further supported by a recent review highlighting its role in atherosclerosis progression and other cardiovascular complications of diabetes (Theofilis et al., 2023).

### 3.5 miR-126 in CVD: from endothelial homeostasis to thrombotic risk

#### 3.5.1 Atherosclerosis and myocardial infarction (MI)

Atherosclerosis is a chronic inflammatory disease of the arteries, characterized by the formation of atherosclerotic plaques within the vascular lumen. Developing insidiously over decades, it underlies the majority of CVD, which remain the leading cause of death worldwide despite major advances in prevention, diagnosis, and treatment (Theofilis et al., 2023). In recent years, scientific attention has increasingly focused on epigenetic mechanisms, particularly miRNAs, as key regulators of CVD pathogenesis (Theofilis et al., 2023). Among these, miR-126-3p has been extensively studied for its cardiovascular relevance.

Atherosclerosis contributes to CVD pathogenesis by causing partial or complete vascular obstruction, impaired blood flow, and tissue hypoxia, ultimately leading to ischemia, cell death, and necrosis. To prevent ischemic injury and stimulate tissue regeneration, angiogenesis is initiated, involving endothelial cell proliferation, migration, and intercellular signaling to form new blood vessels. Bassand et al. demonstrated that miR-126-3p promotes angiogenesis by activating pro-angiogenic Erk1/2 and PI3K/Akt signaling pathways downstream of the chemokine CXCL12. The miR-126/CXCL12 axis facilitates endothelial cell migration and vascular network formation, suggesting that it may represent a potential therapeutic target in atherosclerosis (Bassand et al., 2021). Autophagy has also gained attention in CVD research. Shi et al. investigated the role of miR-126-3p in regulating autophagy following MI. Autophagy is a critical intracellular mechanism for removing dysfunctional or aging cellular components, but excessive autophagy during MI can be detrimental, leading to degradation of essential proteins and organelles, and ultimately, cardiomyocyte death. Beclin-1, a central regulator of autophagy, has been identified as a direct target of miR-126-3p. Overexpression of miR-126-3p suppresses Beclin-1 activity, thereby reducing excessive autophagy and protecting cardiomyocytes after MI. These findings indicate that miR-126-3p overexpression may represent a cardioprotective mechanism, and modulation of miR-126-3p expression may represent a potential therapeutic approach in MI (Shi et al., 2020).

Clinically, decreased expression of miR-126-3p has been consistently observed in high-risk cardiovascular conditions, including DM and hypertension. Reduced circulating levels have been reported in coronary artery disease (CAD) and MI, and lower miR-126-3p expression is associated with a higher risk of major cardiovascular events. These findings support its potential diagnostic and prognostic value in CVD and suggest that modulation of miR-126-3p expression may have therapeutic relevance (Hromádka et al., 2021).

#### 3.5.2 Platelet activation and thrombotic risk

Hromádka et al. conducted a study involving 598 MI patients undergoing primary percutaneous coronary intervention (PCI) to evaluate the relationship between miR-126-3p, platelet activation, and subsequent ischemic events. They demonstrated that miR-126-3p, in combination with miR-223-3p, may serve as predictive biomarker candidates for recurrent thrombotic events in post-MI patients (Hromádka et al., 2021). While serum miR-223-3p primarily originates from platelets and megakaryocytes, miR-126-3p is highly expressed in endothelial cells (ECs) and is also detectable in platelets (Hromádka et al., 2021).

A combined pattern of reduced miR-126-3p and elevated miR-223-3p expression was associated with a higher incidence of recurrent cardiovascular events after MI. Accordingly, the ratio of these two miRNAs may represent a novel parameter for thrombotic risk stratification. Importantly, no significant correlation was observed between these miRNAs and bleeding complications, suggesting that they may function as independent predictors of recurrent ischemic events in multivariable analysis (Hromádka et al., 2021).

### 3.6 miR-126 in chronic kidney disease (CKD): linking endothelial dysfunction to fibrotic progression

#### 3.6.1 Endothelial dysfunction and fibrosis

CKD, along with CVD, hypertension, obesity, and DM, is recognized as one of the major non-communicable diseases of the 21st century. It represents a substantial global health burden, affecting approximately 13.4% of the world’s population, with most patients diagnosed at advanced stages (Evans et al., 2022). CKD contributes significantly to global mortality and remains strongly associated with cardiovascular complications, which account for the majority of deaths in this population.

A major contributor to the high morbidity and mortality associated with CKD is low disease awareness among both patients and healthcare providers. Early stages are typically asymptomatic, and without timely intervention, CKD progresses toward end-stage renal disease accompanied by systemic vascular dysfunction and multi-organ complications. Therefore, improving early detection and identifying reliable molecular biomarker candidates are essential for enabling timely therapeutic intervention.

At the mechanistic level, miR-126 plays a pivotal role in maintaining endothelial integrity and vascular homeostasis—two processes profoundly disrupted in CKD. Experimental evidence from a unilateral ureteral obstruction (UUO) model demonstrates that miR-126 modulates macrophage polarization and regulates renal inflammatory responses through inhibition of the PI3K/insulin receptor substrate-1 (IRS-1)/focal adhesion kinase (FAK) signaling axis, highlighting its context-dependent and potentially maladaptive involvement in fibrotic remodeling and immune cell activation in obstructive nephropathy (Luo X. et al., 2024). These findings underscore the dual role of miR-126 as both a vascular regulator and a mediator of inflammatory–fibrotic signaling.

In addition, prospective human data indicate that reduced baseline circulating miR-126 levels were associated with a more rapid decline in kidney function over time in a cohort of adults without CKD at baseline, suggesting that dysregulation of miR-126 may precede overt functional deterioration (Fujii et al., 2021). Together, these observations position miR-126 at the intersection of endothelial dysfunction, immune activation, and progressive renal fibrosis.

#### 3.6.2 Biomarker and translational potential

Circulating miR-126-3p levels have frequently been reported to be reduced in patients with advanced CKD and correlate with estimated glomerular filtration rate (eGFR) and selected hematologic and metabolic parameters, supporting their potential diagnostic and prognostic value (Franczyk et al., 2022).

Notably, data from a large CKD cohort demonstrated that circulating miR-126 levels were associated with renal function (eGFR) and markers of endothelial dysfunction, including Syndecan-1 (Fourdinier et al., 2021).

Beyond simple association, dysregulation of both miR-126-3p and miR-126-5p contributes to microvascular rarefaction and impaired angiogenic capacity—hallmarks of progressive CKD characterized by capillary loss, chronic hypoxia, and fibrotic expansion.

Mechanistically, miR-126-3p targets key negative regulators of pro-angiogenic signaling, including PIK3R2 and SPRED1, thereby enhancing PI3K/AKT and MAPK/ERK pathway activity and supporting endothelial repair. In CKD, persistent endothelial injury and chronic inflammation disrupt this regulatory balance, leading to impaired reparative angiogenesis and accelerated fibrotic remodeling (Franczyk et al., 2022).

Recent evidence further indicates that miR-126 downregulation is associated with vascular calcification and systemic endothelial dysfunction in CKD, reinforcing its role as a molecular bridge between renal and cardiovascular pathology (da Cunha et al., 2025). Restoration of miR-126-related signaling has been suggested as a potential therapeutic strategy in experimental settings, with the aim of protecting renal microvasculature and mitigating cardiovascular risk (da Cunha et al., 2025). Importantly, accumulating data from DKD research indicate that miR-126-3p/5p downregulation represents a common feature of diabetic kidney injury and may overlap with molecular mechanisms observed in non-diabetic CKD phenotypes (Zou et al., 2026). Targeting miR-126-regulated pathways may thus attenuate inflammatory and pro-fibrotic responses in experimental models of diabetic and non-diabetic kidney disease, underscoring the potential translational relevance of miR-126 isoforms in precision nephrology.

**FIGURE 3:**
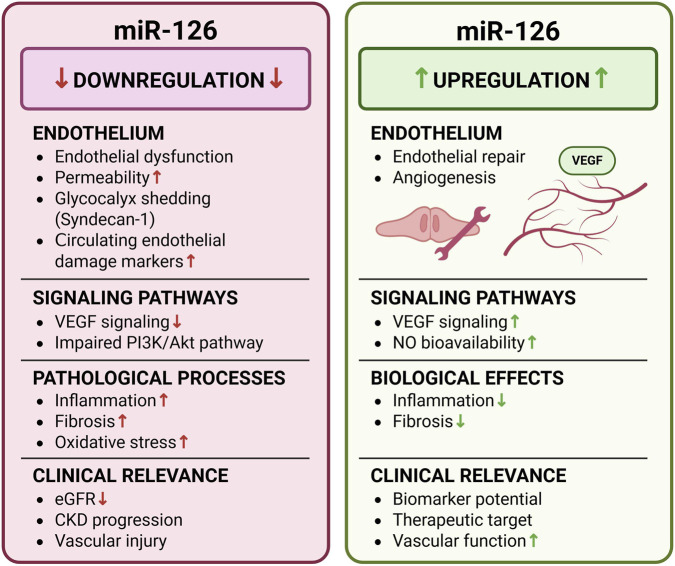
Context-dependent role of miR-126 in CKD. The figure illustrates the dual role of miR-126 under conditions of downregulation and upregulation. Reduced miR-126 expression is associated with endothelial dysfunction, increased vascular permeability, glycocalyx shedding (including Syndecan-1), and elevated circulating markers of endothelial injury. These alterations are associated with impaired VEGF signaling and PI3K/AKT pathway activity, contributing to inflammation, fibrosis, oxidative stress, and decreased eGFR. In contrast, miR-126 upregulation promotes endothelial repair and angiogenesis through activation of VEGF-dependent pathways and improved NO bioavailability. These effects are associated with reduced inflammation and fibrosis, improved vascular function, and support the role of miR-126 as a biomarker candidate and potential therapeutic target in CKD. (Author’s own schematic illustration created with BioRender.com). Abbreviations: AKT – protein kinase B; CKD – chronic kidney disease; eGFR – estimated glomerular filtration rate; NO – nitric oxide; PI3K – phosphoinositide 3-kinase; VEGF – vascular endothelial growth factor.

These findings collectively highlight the context-dependent role of miR-126 in CKD progression, linking endothelial dysfunction with impaired angiogenesis and fibrotic remodeling (Figure 3).

## 4 miR-423-5p: molecular functions and translational relevance

### 4.1 Genomic context and biogenesis

The miR-423 gene is located within an intron of the nuclear speckle splicing regulatory protein 1 (NSRP1) gene, also known as serine/arginine repetitive matrix protein 2 (SRRM2), on chromosome 17q11.2. This intragenic miRNA is processed from the same precursor as miR-423-3p, although both strands may be biologically active, the 5p strand has been more frequently implicated in disease-related contexts (Ke et al., 2020). MiR-423-5p regulates key cellular processes, including apoptosis, cell proliferation, and energy metabolism, primarily through modulation of multiple signaling cascades such as the mitogen-activated protein kinase (MAPK) pathway, transforming growth factor-β (TGF-β) signaling, and PI3K/AKT pathway (Ke et al., 2020). Its activity influences cellular stress responses, metabolic homeostasis, and tissue remodeling, highlighting its pleiotropic role in both physiological and pathological conditions.

### 4.2 miR-423-5p in cancer: dual roles in tumor suppression and oncogenesis

Recent studies have demonstrated that miR-423-5p acts as a context-dependent regulator in multiple malignancies, exhibiting either tumor-suppressive or oncogenic properties depending on the cellular and microenvironmental context.

#### 4.2.1 CRC

In CRC, Jia et al. reported that miR-423-5p expression is significantly reduced in colorectal tumor tissues and cell lines compared with adjacent non-tumorous mucosa (Jia et al., 2018). Functional gain-of-function experiments revealed that forced overexpression of miR-423-5p suppresses proliferation and colony formation while promoting apoptosis in CRC cells. Mechanistically, miR-423-5p–induced apoptosis was caspase-dependent, supported by increased activation of caspase-3/8/9 and attenuation of the effect by a pan-caspase inhibitor, indicating a direct pro-apoptotic role of miR-423-5p in CRC models (Jia et al., 2018). Follow-up evidence further supported this concept by showing that miR-423-5p restoration promotes apoptosis and reverses radioresistance in colorectal cancer models, reinforcing its tumor-suppressive role (Shang et al., 2021).

#### 4.2.2 Prostate, gastric, lung, ovarian, and brain tumors

Beyond CRC, miR-423-5p has been implicated in multiple cancers with predominantly oncogenic roles.

In prostate cancer (PC), Shan et al. showed that cancer-associated fibroblasts (CAFs) secrete exosomes enriched in miR-423-5p, which are internalized by tumor cells and reprogram them toward a more aggressive, chemoresistant phenotype (Shan et al., 2020). Specifically, exosomal miR-423-5p enhances resistance to taxane-based chemotherapy by modulating the TGF-β signaling pathway and directly targeting Gremlin 2 (GREM2), a bone morphogenetic protein (BMP) antagonist involved in stromal–tumor communication. Independently, integrative miRNA–mRNA network analyses identified miR-423-5p as a driver of prostate cancer progression through suppression of Gene associated with Retinoid-Interferon-Induced Mortality 19 (GRIM-19), a mitochondrial regulator with tumor-suppressive properties (Lin et al., 2019). Loss of GRIM-19 activity in response to miR-423-5p supports sustained proliferation and survival signaling, underscoring the oncogenic function of this miRNA in PC (Lin et al., 2019).

In gastric cancer (GC), miR-423-5p expression is downregulated in tumor tissues, and its functional suppression contributes to enhanced proliferation, migration, and invasion through sponging by long non-coding RNA (lncRNA) telomerase RNA component (TERC). Mechanistically, TERC acts as a competing endogenous RNA, sequestering miR-423-5p and thereby upregulating SRY-box transcription factor 12 (SOX12) expression, which promotes tumor progression (Wang J. et al., 2022).

Similarly, in lung adenocarcinoma (LUAD), miR-423-5p downregulates cell adhesion molecule 1 (CADM1), a tumor-suppressive cell-adhesion molecule. Reduced CADM1 expression enhances proliferation, migration, and invasion, indicating that miR-423-5p actively promotes malignant progression in LUAD (Huang and Feng, 2021). Interestingly, miR-423-5p may behave as a tumor suppressor in ovarian cancer (OC). Reduced expression of miR-423-5p in tumor tissue and patient serum correlates with advanced stage and metastatic spread (Wang et al., 2022). Wang et al. (2022) demonstrated that dysregulation of the long non-coding RNA colorectal neoplasia differentially expressed (CRNDE) alters the miR-423-5p/fascin actin-bundling protein 1 (FSCN1) axis, where CRNDE sequesters miR-423-5p, relieving repression of FSCN1, a cytoskeletal regulator. This promotes cell motility and invasion, indicating that loss of functional miR-423-5p signaling contributes to ovarian cancer progression (Wang et al., 2022).

In glioblastoma (GBM), one of the most aggressive adult brain tumors, miR-423-5p is upregulated and promotes tumor growth and therapeutic resistance (Mahinfar et al., 2022). Mechanistically, miR-423-5p suppress key tumor suppressors, a central inhibitor of the PI3K/AKT pathway, enabling persistent survival and proliferation signaling. Increased miR-423-5p confer resistance to temozolomide, the standard chemotherapeutic agent for GBM, highlighting its potential as a therapeutic target candidate for overcoming chemoresistance (Mahinfar et al., 2022).

Taken together, these findings position miR-423-5p as a highly dynamic regulator at the intersection of tumor proliferation, apoptosis, stromal reprogramming, metastatic behavior, and drug resistance. Its context-dependent behavior — tumor-suppressive in CRC, potentially protective in OC, but pro-oncogenic in prostate, gastric, lung, and brain tumors — underscores its dual clinical relevance as both a circulating biomarker and a candidate for pathway-directed therapeutic intervention (Jia et al., 2018; Shang et al., 2021; Lin et al., 2019; Wang J. et al., 2022; Mahinfar et al., 2022).

### 4.3 miR-423-5p in cardiovascular and renal diseases: from mechanisms to clinical applications

#### 4.3.1 Heart failure (HF)

The first description of miR-423-5p in clinical settings concerned its role in HF. Tijsen et al. reported significantly elevated serum miR-423-5p levels in HF patients compared with healthy controls, indicating its potential as a circulating biomarker candidate for differentiating HF from other causes of dyspnea (Tijsen et al., 2010; Yang et al., 2021). Yang et al. performed a systematic review and meta-analysis confirming the potential diagnostic and prognostic value of circulating miR-423-5p in HF, including associations with established clinical parameters (Yang et al., 2021).

In a mechanistic study, Xu et al. investigated the effect of miR-423-5p on angiotensin II (Ang II)–induced cardiomyocyte hypertrophy. They found that Ang II stimulation upregulated miR-423-5p in hypertrophic cardiomyocytes and identified suppressor of Ty6 homolog (SUPT6H) as a direct target. Experimental modulation revealed that downregulation of miR-423-5p attenuated cardiomyocyte hypertrophy *in vitro*, suggesting a context-dependent regulatory role of miR-423-5p in Ang II–induced cardiac remodeling (Xu et al., 2023).

#### 4.3.2 Diabetic nephropathy (DN) and retinopathy (DR)

MiR-423-5p has also been implicated in diabetic microvascular complications. Xu et al. demonstrated reduced miR-423-5p expression and increased NADPH oxidase 4 (Nox4) in kidney tissue from DN patients and in experimental models. High glucose conditions upregulated Nox4 while suppressing miR-423-5p in cultured podocytes. Overexpression of miR-423-5p enhanced podocyte viability, reduced reactive oxygen species (ROS) production, and inhibited apoptosis, inflammation, and cytoskeletal injury. Functional assays confirmed that Nox4 is a direct target of miR-423-5p, highlighting its protective role against hyperglycemia-induced podocyte injury (Xu et al., 2018).

Similarly, Hou et al. demonstrated that miR-423-5p exerts renoprotective effects through the miR-423-5p/upstream stimulating factor 2 (USF2) axis in DN. The study focused on apigenin (APG), a flavonoid with anti-inflammatory, antioxidant, and antifibrotic properties. In DN, miR-423-5p is downregulated, while USF2 is upregulated, accelerating disease progression. Apigenin treatment restored miR-423-5p expression, suppressed USF2, and improved podocyte structure and function, suggesting that modulation of the miR-423-5p/USF2 axis may represent a potential therapeutic approach in experimental models of DN (Hou et al., 2021). Circulating microRNAs have also been investigated as non-invasive biomarkers for diabetic retinopathy, with recent systematic evidence supporting their potential diagnostic value across different disease stages (Martínez-Santos et al., 2025). Emerging data also highlight the role of miR-423-5p in diabetic retinopathy (DR). Liu et al. demonstrated that miR-423-5p promotes Müller cell activation and retinal microvascular dysfunction by targeting NGF signaling. In diabetic mice, miR-423-5p was upregulated in retinal tissue and circulation, contributing to oxidative stress and inflammatory responses (Liu et al., 2023).

Xiao et al. further investigated neovascularization mechanisms in DR, focusing on the E2F transcription factor 1 (E2F1)/miR-423-5p/Homeodomain-Interacting Protein Kinase 2 (HIPK2) axis. In this study, E2F1 was shown to transcriptionally activate miR-423-5p, which directly targets HIPK2, a kinase that restrains hypoxia-inducible factor 1 alpha (HIF-1α)/VEGF signaling. Suppression of HIPK2 led to disinhibition of the HIF-1α/VEGF pathway, thereby promoting retinal angiogenesis. These findings provide mechanistic insights into DR pathogenesis and identify potential molecular targets for further therapeutic investigation (Xiao et al., 2021).

Notably, these divergent effects underscore the context-dependent behavior of miR-423-5p across distinct diabetic microvascular complications.

### 4.4 Circulating miR-423-5p as a reference and biomarker

Beyond its disease-specific roles, miR-423-5p has been evaluated as a stable circulating microRNA in CKD. Wong et al. analyzed serum miRNA profiles in patients with CKD of various etiologies, including individuals undergoing hemodialysis, to assess the stability of candidate reference miRNAs (Wong et al., 2023).

Among the evaluated molecules, miR-423-5p demonstrated one of the highest expression stability indices across different eGFR strata and clinical subgroups. In contrast to several other circulating miRNAs that exhibited significant variability, miR-423-5p remained relatively constant, supporting its potential suitability as an endogenous normalization control in circulating miRNA studies in CKD (Guo et al., 2025).

These findings are particularly relevant for translational research, where accurate normalization is critical for reliable quantification of circulating miRNAs. Thus, in addition to its context-dependent biological functions, miR-423-5p may serve a methodological role in biomarker development.

## 5 Translational horizons: emerging therapeutic and diagnostic roles of miR-126-3p, miR-126-5p, and miR-423-5p

To provide a comprehensive overview of the overlapping and distinct roles of miR-126-3p, miR-126-5p, and miR-423-5p across cancer, cardiovascular, and renal diseases, a comparative summary is presented in Table 1. This integrative table highlights the shared and unique molecular mechanisms of these miRNAs, emphasizing their involvement in endothelial function, oxidative stress, inflammation, angiogenesis, and fibrotic remodeling. It details tissue and cell specificity, key molecular targets and signaling pathways, direction of dysregulation in various disease contexts, primary disease associations, and their diagnostic or therapeutic significance.

**TABLE 1:** Comparative overview of miR-126-3p, miR-126-5p, and miR-423-5p: biological roles, signaling pathways, and clinical relevance.

miRNA	Tissue/Cell type	Key targets and pathways	Direction of change (disease context)	Main diseases	Diagnostic/Therapeutic significance
miR-126-3p	Endothelium, platelets, endothelial progenitor cells (EPCs)	PIK3R2, SPRED1 → PI3K/AKT and ERK-driven angiogenic signaling	Generally ↓ in diabetes and CKD; frequently downregulated in cancer (tumor-type dependent)	DKD, CVD, NSCLC, BC, HCC, CRC	Promotes endothelial repair and angiogenesis; investigational biomarker of endothelial dysfunction; potential therapeutic target for vascular protection
miR-126-5p	Endothelium, vascular smooth muscle cells	VCAM1, CRK → MAPK and NF-κB-associated signaling	Frequently dysregulated in metabolic and vascular injury (direction context-dependent)	CKD, CVD, selected cancers	Associated with anti-inflammatory and vascular-protective effects; investigational therapeutic target in endothelial and vascular injury
miR-423-5p	Renal tubular cells, cardiomyocytes, cancer cells	NOX4, USF2, SUPT6H → TGF-β, ROS, and insulin-related signaling pathways	Reported ↓ in experimental DKD models; ↑ in heart failure; context-dependent dysregulation in cancer	DN/DKD, CVD, PC, GC, GBM	Associated with oxidative-stress regulation and cardiac remodeling; validated stable reference miRNA for normalization in CKD studies; context-dependent biomarker and potential therapeutic target

Abbreviations: BC, breast cancer; CKD, chronic kidney disease; CRC, colorectal cancer; CVD, cardiovascular disease; DKD, diabetic kidney disease; DN, diabetic nephropathy; EPCs, endothelial progenitor cells; GBM, glioblastoma multiforme; GC, gastric cancer; HCC, hepatocellular carcinoma; NSCLC, non-small-cell lung cancer; PC, prostate cancer; PIK3R2, phosphoinositide-3-kinase regulatory subunit 2; SPRED1 – sprouty-related EVH1 domain-containing protein 1; PI3K, phosphoinositide 3-kinase; AKT, protein kinase B; ERK, extracellular signal-regulated kinase; MAPK, mitogen-activated protein kinase; VEGF, vascular endothelial growth factor; VCAM1, vascular cell adhesion molecule 1; CRK–CT10, regulator of kinase; NF-κB, nuclear factor kappa-light-chain-enhancer of activated B cells; NOX4 – NADPH oxidase 4; USF2, upstream stimulating factor 2; SUPT6H, suppressor of Ty 6 homolog; TGF-β, transforming growth factor beta; ROS, reactive oxygen species. Source: Author’s own elaboration based on references cited in Sections 3–5.

**FIGURE 4:**
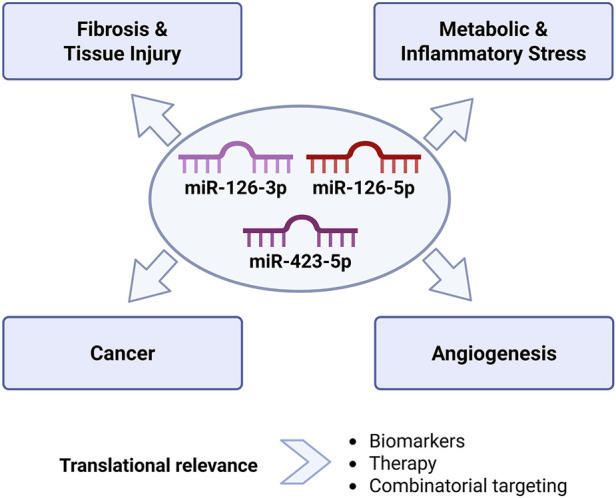
Integrative roles of miR-126-3p, miR-126-5p, and miR-423-5p in metabolic, cardiovascular and oncological diseases. MiR-126-3p, miR-126-5p, and miR-423-5p form an interconnected regulatory network linking endothelial function, metabolic homeostasis, inflammation, fibrotic remodeling, and tumor biology. Through coordinated modulation of shared pathophysiological processes, these miRNAs influence vascular integrity, oxidative stress, tissue remodeling, and disease progression across metabolic, cardiovascular, renal, and oncological disorders. This integrative framework highlights their translational relevance as biomarkers and therapeutic targets in precision medicine. (Author’s own schematic illustration created with BioRender.com).

To complement the tabular data, Figure 4 provides a conceptual schematic of the coordinated and disease-overlapping roles of miR-126-3p, miR-126-5p, and miR-423-5p. The figure illustrates their cell- and tissue-specific targets, downstream signaling cascades, and functional outcomes across multiple pathological contexts, offering a visual framework for understanding their translational potential as biomarkers and therapeutic modulators. Together, Table 1 and Figure 4 provide an integrative platform for contextualizing the mechanistic and clinical relevance of these miRNAs in disease pathophysiology.

### 5.1 miRNA-based diagnostics: liquid biopsies and biomarker panels

Both strands derived from the miR-126 precursor—miR-126-3p and miR-126-5p—are increasingly recognized as complementary regulators of vascular biology and metabolic disease, with endothelial-enriched expression and links to angiogenic signaling and endothelial integrity (Guo et al., 2025). In clinical studies across diabetes populations, circulating miR-126 shows a reproducible tendency toward dysregulation, suggesting its potential role as a minimally invasive biomarker candidate in cardiometabolic risk stratification, although the available evidence remains heterogeneous and does not yet support its use as a definitive biomarker (Athira et al., 2022). Importantly for nephrology, liquid-biopsy approaches based on urinary and exosome-associated miRNAs are actively being developed for DKD, leveraging the stability of miRNAs in urine and urinary extracellular vesicles and enabling integration with conventional markers such as urinary albumin-to-creatinine ratio (UACR) and eGFR (Lee et al., 2023).

In contrast to the endothelium-restricted expression pattern typically emphasized for miR-126, miR-423-5p has been investigated in kidney disease both as a biologically relevant miRNA and as a technically useful molecule in translational workflows. Mechanistically, miR-423-5p has been linked to oxidative stress regulation in DKD via Nox4-centered pathways, with downstream consequences for podocyte injury, reactive oxygen species generation, and inflammatory signaling (Wang D. et al., 2023). In addition, experimental studies indicate that the natural flavonoid apigenin can exert renoprotective effects in diabetic nephropathy models through the miR-423-5p–upstream stimulatory factor 2 (USF2) axis, connecting microRNA regulation with anti-inflammatory and anti-fibrotic outcomes (Hou et al., 2021).

Beyond its functional roles, miR-423-5p has also been evaluated in the context of methodological considerations in circulating miRNA research, including observations of relatively stable expression patterns, suggesting potential utility in normalization strategies for circulating miRNA studies (Guo et al., 2023). Taken together, these findings suggest a pragmatic translational framework in which miR-126 (strand-aware where possible) and miR-423-5p may be incorporated into liquid-biopsy panels and interpreted alongside standard clinical parameters to enhance early detection and monitoring strategies in DKD. However, the clinical utility of circulating miR-126 remains to be fully established, and further validation in large, well-designed prospective studies is required.

### 5.2 Therapeutic strategies and combinatorial modulation

Several therapeutic strategies are being explored to restore or modulate protective microRNAs in complex cardio-metabolic and renal diseases. Advances in RNA biology and delivery technologies have substantially expanded the translational landscape of miRNA-based interventions.

#### 5.2.1 Exosome-based delivery

Extracellular vesicles, including exosomes, are increasingly recognized as biologically active carriers capable of transferring regulatory RNAs between cells. Their intrinsic biocompatibility, capacity for tissue targeting, and ability to protect RNA cargo from degradation make them attractive candidates for therapeutic delivery platforms (Kalluri and LeBleu, 2020). Experimental engineering approaches allow selective loading of small RNAs into extracellular vesicles, enabling modulation of inflammatory and fibrotic signaling pathways in preclinical models. Although clinical translation remains at an early stage, exosome-based systems represent a promising avenue for organ-directed RNA therapy.

#### 5.2.2 Nanoparticle and chemically modified oligonucleotide systems

Efficient delivery remains a central challenge in RNA therapeutics. Advances in oligonucleotide chemistry, lipid nanoparticle formulations, and conjugation strategies have significantly improved molecular stability, tissue uptake, and pharmacokinetic profiles of RNA-based drugs (Roberts et al., 2020). These developments have facilitated the clinical advancement of multiple RNA therapeutics and provide a technological framework for future miRNA mimic and inhibitor strategies in renal and metabolic diseases.

#### 5.2.3 Regulatory RNA networks (ceRNA layer)

Therapeutic modulation of miRNAs must account for the complexity of post-transcriptional regulatory networks. Long non-coding RNAs (lncRNAs) and other RNA species dynamically regulate miRNA availability through competing endogenous RNA (ceRNA) mechanisms, thereby influencing gene-expression landscapes across tissues (Statello et al., 2023). This multilayered regulatory architecture may contribute to disease heterogeneity and influence therapeutic responsiveness, underscoring the importance of network-aware design strategies in miRNA-based interventions.

#### 5.2.4 miRNA mimics, inhibitors, and combinatorial modulation

Synthetic miRNA mimics and inhibitors remain core tools in preclinical therapeutic development. Beyond single-miRNA targeting, combinatorial strategies are emerging as a means to enhance biological efficacy. Experimental studies demonstrate that simultaneous loading of multiple microRNAs into extracellular vesicles can amplify anti-inflammatory effects compared with single-miRNA approaches, highlighting the potential of network-based therapeutic designs (Pottash et al., 2022). Such combinatorial modulation may be particularly relevant in multifactorial diseases characterized by intertwined inflammatory, metabolic, and fibrotic pathways.

Collectively, these advances emphasize that successful translation of miRNA modulation into clinical practice will depend not only on identifying protective or pathogenic miRNAs but also on optimizing delivery platforms and accounting for complex RNA regulatory networks.

**FIGURE 5:**
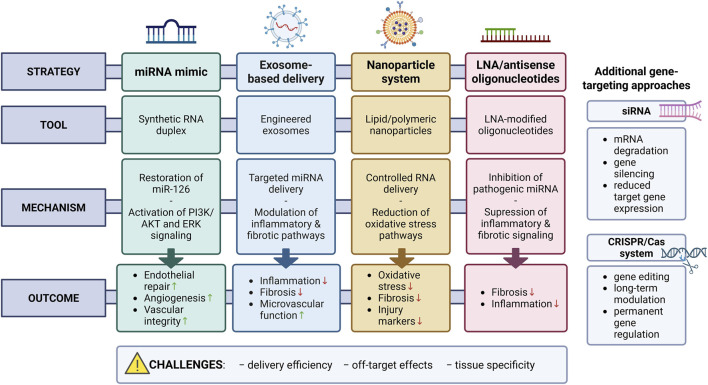
Experimental strategies for miRNA modulation in kidney disease. The figure schematically summarizes the main preclinical approaches for modulating miRNA expression, including miRNA mimics, antisense oligonucleotides (LNA-based inhibitors), exosome-based delivery, and lipid/polymeric nanoparticle systems. Additional gene-targeting approaches, such as siRNA and CRISPR/Cas-based systems, are also presented. For each strategy, the corresponding molecular tools, mechanisms of action, and representative biological outcomes are illustrated. These approaches aim to restore protective miRNAs or inhibit pathogenic ones, thereby modulating key pathways involved in inflammation, fibrosis, oxidative stress, and microvascular dysfunction. Collectively, these strategies highlight the translational potential of miRNA-based therapies, emphasizing the importance of delivery efficiency, tissue specificity, and minimization of off-target effects in future clinical applications. (Author’s own schematic illustration created with BioRender.com). Abbreviations: AKT – protein kinase B; ERK – extracellular signal-regulated kinase; LNA – locked nucleic acid; PI3K – phosphoinositide 3-kinase; siRNA – small interfering RNA.

The main experimental approaches and delivery systems for modulating miRNA expression in preclinical models are summarized in Table 2 and illustrated schematically in Figure 5. These strategies aim to restore protective miRNAs, including miR-126-3p, miR-126-5p, and miR-423-5p, in diabetic and metabolic kidney disease. They outline the molecular tools and delivery platforms designed to regulate vascular, inflammatory, and fibrotic pathways. Preclinical evidence indicates that these interventions can enhance endothelial repair, reduce oxidative stress, and improve renal microvascular integrity.

**TABLE 2:** Preclinical Therapeutic Strategies for Modulating miRNA Expression in Diabetic and Metabolic Kidney Disease.

Therapeutic strategy	Molecular tool/Vehicle	Target miRNA(s)	Mechanism of action	Preclinical outcome
miRNA mimic	Synthetic double-stranded RNA duplex	miR-126-3p, miR-126-5p	Restoration of endothelial-enriched miR-126 strands; derepression of PIK3R2/SPRED1 and enhancement of PI3K/AKT and ERK-driven angiogenic signaling	Improved endothelial repair and angiogenic capacity; enhanced vascular integrity and microvascular function (preclinical)
Exosome-based delivery	Engineered extracellular vesicles (exosomes)	miR-126-3p (± other protective miRNAs; investigational)	Targeted renal/endothelial delivery of miRNA cargo; modulation of inflammatory, oxidative-stress and pro-fibrotic signaling	Attenuation of inflammatory and fibrotic responses; improved microvascular function in experimental models
Nanoparticle system	Lipid or polymeric nanoparticle (e.g., PLGA-, PEG-based)	Therapeutic miRNA mimic/inhibitor (cargo-dependent; preclinical)	Controlled release and tissue uptake of RNA therapeutics; suppression of oxidative-stress and pro-fibrotic pathways depending on cargo	Reduced oxidative stress and fibrotic remodeling; improved injury markers in preclinical models
LNA-based inhibitors/antisense oligonucleotides	LNA-modified oligonucleotides	Pathogenic miRNAs (context-dependent; preclinical)	LNA/ASO-mediated inhibition of miRNA function; reduction of downstream inflammatory and fibrotic signaling	Reduced fibrosis and inflammatory activation in experimental renal/cardiac models (miRNA- and context-dependent)

Abbreviations: AKT, protein kinase B; DKD, diabetic kidney disease; LNA, locked nucleic acid; PEG, polyethylene glycol; PI3K, phosphoinositide 3-kinase; PLGA, poly(lactic-co-glycolic acid); RNA, ribonucleic acid; ROS, reactive oxygen species; VEGF, vascular endothelial growth factor. Source: Author’s own elaboration based on references cited in Sections 3–5.

### 5.3 Translational potential and clinical implementation

The coming decade may witness increasing clinical integration of miRNA-based diagnostics and selected therapeutic strategies. Integration of miR-126-3p, miR-126-5p, and miR-423-5p into liquid-biopsy panels, alongside traditional biomarkers, may enhance early detection, risk assessment, and treatment monitoring.

Advances in RNA chemical stabilization, exosome engineering, nanoparticle delivery, and AI-driven bioinformatics are poised to accelerate the development of organ-targeted, safe, and efficient miRNA therapeutics.

MiR-126-3p, miR-126-5p, and miR-423-5p represent promising candidate microRNAs, bridging endothelial repair, antifibrotic protection, and metabolic regulation. Their integration into personalized diagnostic and therapeutic frameworks represents a potential step toward molecularly guided treatment in nephrology, cardiology, and metabolic medicine (Rodzoń-Norwicz et al., 2025; Pottash et al., 2022).

Taken together, miR-126 and miR-423-5p emerge as context-dependent regulators operating at the intersection of endothelial function, inflammation, oxidative stress, and tissue remodeling. While miR-126 is primarily linked to endothelial homeostasis and angiogenic signaling, miR-423-5p appears to integrate stress-response pathways and metabolic adaptation across different organ systems. Importantly, both microRNAs exhibit dynamic, disease-specific expression patterns, reflecting the complexity of their regulatory networks.

From a translational perspective, their combined evaluation may provide a more comprehensive insight into disease progression, particularly in cardiometabolic and renal disorders, where vascular dysfunction and fibrosis coexist. However, their clinical applicability remains limited by heterogeneity of available data, lack of standardization, and insufficient prospective validation.

## 6 Limitations and challenges

Despite significant advances in understanding the role of miRNAs in chronic and diabetic kidney disease, several limitations continue to constrain their clinical translation.

The first challenge is the lack of methodological standardization across studies. Diverse platforms for miRNA quantification (e.g., RT-qPCR, microarrays, and next-generation sequencing), combined with varying biological matrices (plasma, serum, urine, exosomes), generate heterogeneous and often incomparable results. Garmaa et al. emphasized that inconsistencies in RNA extraction, normalization, and analytical pipelines substantially limit reproducibility in CKD and DKD studies, complicating the identification of robust biomarker panels (Garmaa et al., 2024).

A second limitation involves the absence of universally accepted endogenous controls for normalization in kidney-related miRNA studies. Previous studies have suggested that miR-423-5p may exhibit relatively stable expression across CKD stages, indicating its potential utility as an internal reference rather than as a biomarker. However, the lack of validated normalization standards remains a key barrier to harmonized data interpretation (Lakkisto et al., 2023).

The third challenge concerns biological and clinical variability. Factors such as patient age, diabetes duration, treatment exposure, comorbidities, and sample timing can profoundly affect miRNA levels. Motshwari et al. highlighted that heterogeneity in study design and patient phenotypes undermines the comparability of findings, impeding the establishment of clinically meaningful diagnostic thresholds (Motshwari et al., 2023).

Therapeutic delivery and specificity constitute another major obstacle. Although exosome- and nanoparticle-based carriers have improved kidney-targeted miRNA transport, issues such as limited bioavailability, off-target effects, suboptimal tissue penetration, and potential immunogenicity persist. Momin et al. emphasized that despite encouraging preclinical efficacy of miRNA mimics and inhibitors, substantial challenges remain regarding pharmacokinetics, molecular stability, targeted delivery, and long-term safety, which continue to hinder clinical translation (Momin et al., 2021).

Finally, regulatory, ethical, and translational hurdles must be addressed before miRNA-based diagnostics and therapeutics can reach clinical practice. Cao et al. emphasized that large-scale validation trials, standardized protocols, and advanced delivery platforms (including AI-optimized exosome engineering and RNA chemical stabilization) are essential for ensuring reproducibility and safety (Cao et al., 2025).

While miR-126-3p, miR-126-5p, and miR-423-5p exhibit potential diagnostic and therapeutic relevance, future research should prioritize multi-center validation, standardization of analytical methods, and integration of multi-omics data to accelerate their translation into precision medicine.

Furthermore, integrating the context-dependent roles of miR-126 and miR-423-5p across different disease systems may support the development of more precise diagnostic and therapeutic strategies.

## 7 Conclusion

This review highlights the multifaceted biological and clinical significance of miR-126-3p, miR-126-5p, and miR-423-5p across cancer, cardiovascular, metabolic, and kidney diseases. Accumulating evidence demonstrates that these miRNAs play pivotal roles in regulating endothelial homeostasis, inflammatory responses, oxidative stress, and fibrotic remodeling—key mechanisms underlying disease initiation and progression in cardio-metabolic and renal disorders.

From a translational perspective, miR-126-3p and miR-126-5p emerge as central regulators of vascular integrity and endothelial repair, whereas miR-423-5p acts as a context-dependent modulator of cellular stress responses and fibrotic signaling. Their remarkable stability in biological fluids, particularly in circulating and exosomal fractions, supports their utility as non-invasive biomarkers for early disease detection, risk stratification, and therapeutic monitoring.

Moreover, growing preclinical evidence suggests that restoration of physiological miRNA levels through miRNA mimics, exosome-based delivery systems, or nanoparticle platforms may attenuate vascular and renal injury, underscoring their therapeutic potential.

Future research should focus on integrating miRNA-based biomarkers into multimodal diagnostic strategies, combining liquid biopsy approaches with established clinical parameters. Advances in RNA chemical stabilization, targeted delivery technologies, and systems biology are expected to accelerate the clinical translation of miRNA-based diagnostics and therapeutics. Equally important is a deeper understanding of miRNA interactions within complex non-coding RNA networks and disease-specific regulatory circuits, which will enable the development of precision, phenotype-tailored interventions.

In conclusion, miR-126-3p, miR-126-5p, and miR-423-5p represent a promising class of clinically relevant miRNAs that bridge molecular mechanisms with translational applications. Their integration into personalized diagnostic and therapeutic frameworks has the potential to significantly advance precision medicine in nephrology, cardiovascular care, and metabolic disease management.
